# Seasonal Dynamics, Botanical Origin, and Yield of *Apis mellifera* Propolis in the Mexican Altiplano: Influence of Precipitation, Phenology, and Harvesting Methods

**DOI:** 10.3390/insects17101013

**Published:** 2026-10-01

**Authors:** Jose Juan Alcivar-Saldaña, Marco Aurelio Rodriguez-Monroy, Lysett Corona-Gómez, Víctor Manuel Díaz-Sánchez, Manuel Andrés González-Toimil, Noe Garcia-Cedillo, Maria Margarita Canales-Martinez

**Affiliations:** 1 Departamento de Ciencias Pecuarias, Facultad de Estudios Superiores Cuautitlán, Universidad Nacional Autónoma de México (UNAM), Carretera Cuautitlán-Teoloyucan km. 2.5, Colonia San Sebastián Xhala, Cuautitlán Izcalli C.P. 54714, Estado de Mexico, Mexico; bio.alcivar@gmail.com (J.J.A.-S.); victordiaz@cuautitlan.unam.mx (V.M.D.-S.); 2 Laboratorio de Investigación Biomédica en Productos Naturales, Carrera de Medicina, Facultad de Estudios Superiores Iztacala, Universidad Nacional Autónoma de México (UNAM), Avenida de los Barrios Número 1, Colonia Los Reyes Iztacala, Tlalnepantla de Baz C.P. 54090, Estado de Mexico, Mexico; dr.marcorodriguezmonroy@gmail.com; 3 Departamento de Ciencias Biológicas, Facultad de Estudios Superiores Cuautitlán, Universidad Nacional Autónoma de México (UNAM), Carretera Cuautitlán-Teoloyucan km. 2.5, Colonia San Sebastián Xhala, Cuautitlán Izcalli C.P. 54714, Estado de Mexico, Mexico; lysett.corona@cuautitlan.unam.mx; 4 Centro de Enseñanza Agropecuaria, FES Cuautitlán, Carretera Cuautitlán-Teoloyucan km. 2.5, Colonia San Sebastián Xhala, Cuautitlán Izcalli C.P. 54714, Estado de Mexico, Mexico; toimil@comunidad.unam.mx; 5 Subdirección de Impulso a la Producción Acuícola, Avícola y Apícola, Secretaria del Campo (SECAMPO), Conjunto SEDAGRO Rancho San Lorenzo S/N, Manzana 001, San Lorenzo, Coacalco C.P. 52140, Estado de México, Mexico; biocolced@gmail.com; 6 Laboratorio de Farmacognosia, Unidad de Biotecnología y Prototipos (UBIPRO), Facultad de Estudios Superiores Iztacala, Universidad Nacional Autónoma de México (UNAM), Avenida de los Barrios Número 1, Colonia Los Reyes Iztacala, Tlalnepantla de Baz C.P. 54090, Estado de Mexico, Mexico

**Keywords:** propolis, *Apis mellifera*, melissopalynology, plant phenology, botanical origin

## Abstract

Propolis yield and composition fluctuate with environmental factors and hive management. In a two-year longitudinal study (2022–2024), we evaluated the seasonal dynamics of propolis production across two apiaries in the Mexican Altiplano during the dry and rainy seasons. We conducted monthly harvesting using three collector types: black polyurethane collectors (G1), beige polyurethane collectors (G2), and flexible polyethylene mosquito mesh (G3), distributed among 24 standardized *Apis mellifera* colonies (*n* = 8 per treatment; 576 repeated monthly harvests). Palynological analysis identified 39 species belonging to 38 genera and 23 families, with a clear predominance of herbaceous and ruderal forms (*Brassica rapa* as the main pollen type). The overall mean yield was 9.02 ± 0.58 g/colony/month. Linear mixed-effects models (LMMs) revealed significant temporal variation by month (*F*_11_,_231_ = 28.45, *p* < 0.001) and season (*F*_1_,_21_ = 14.12, *p* = 0.001). Propolis biomass showed a clear bimodal pattern, peaking in April (20.25 g/colony) during vegetative bud burst, dropping in July (6.25 g/colony), and rebounding in September–October (9.77–9.92 g/colony), coinciding with leaf senescence and pre-winter nest sealing. Monthly production showed a moderate negative linear correlation with cumulative precipitation (*r* = −0.422, *p* = 0.040, *R*^2^ = 0.178). Conversely, collector design did not result in statistically significant differences in harvested biomass (G1: 6.88 g, G2: 8.98 g, G3: 11.21 g; *F*_2_,_21_ = 2.14, *p* = 0.142). These findings demonstrate that propolis collection in the Mexican Altiplano is primarily synchronized with regional plant phenology and constrained by precipitation regimes rather than collector design. Consequently, the practical selection of harvesting devices should be guided by operational handling efficiency, economic cost, and raw product cleanliness, rather than expectations of higher quantitative yield.

## Simple Summary

This study analyzes how propolis production by honeybees varies throughout the year across the dry and rainy seasons in the Mexican Altiplano, considering environmental factors such as rainfall and plant cycles. Over two years, we evaluated beehives in two apiaries using two types of collectors and identified pollen from plants the bees visited. The results showed that propolis harvest varied significantly by month and season, peaking in April—coinciding with plant bud burst—and showing a second, smaller increase between September and October, related to leaf fall and hive preparation for winter. Conversely, rainfall showed a moderately negative relationship with production, whereas the type of collector used did not significantly affect yield. These findings show that propolis collection depends mainly on natural vegetation cycles and local climate, helping beekeepers choose tools based on convenience and cost rather than expecting higher yields from collector design.

## 1. Introduction

It has been widely documented that propolis contains a wide range of chemical components, including polyphenols, flavonoids, terpenes, vitamins, and minerals, which confer a broad spectrum of biological properties, such as antimicrobial, antioxidant, anti-inflammatory, and cardioprotective effects. However, it should be noted that this chemical composition is highly variable, as it depends on biotic and abiotic factors in the production area [1,2,3,4,5]. For this reason, propolis production methods are of particular importance, as they significantly influence both the quantity and quality of the propolis obtained. In turn, these methods vary according to each production area and season [6,7].

Seasonality, for its part, considerably influences propolis production, because a high percentage of the raw materials required are collected from the flower buds of various tree species, as well as from balms from the axillary regions of certain herbaceous plants. Each of these groups exhibits distinct production patterns that are governed by abiotic factors, such as rainfall periods, during which a higher number of visits by *Apis mellifera* have been observed as they collect these resources, suggesting possible resin flows similar to nectar flows [8,9,10,11].

In Mexico, seasonal variation is pronounced, as precipitation levels delineate two distinct regimes: the dry season and the rainy season. Each period is characterized by the vegetative growth and flowering of specific plant communities across the country’s beekeeping regions. In the Mexican Altiplano, these marked seasonal fluctuations directly impact floral resource availability, bud break, and consequently, the volume of propolis collected by honey bees [2,8,10].

Methods of modernizing propolis production processes comprise a set of strategies, structural modifications, and management practices implemented in the hives with the aim of optimizing both the quantity and quality of the propolis harvested. These methods are based on knowledge of bee ethology regarding their natural propolis-gathering behavior [6].

We hypothesized that propolis production in the Mexican Altiplano is closely synchronized with seasonal climatic transitions and specific plant phenological phases (i.e., bud burst and overwintering preparation), and that it shows an inverse association with precipitation. In contrast, collector type does not significantly influence total harvested biomass. Therefore, this study aimed to evaluate the seasonal dynamics of propolis yield over two consecutive years in relation to local precipitation patterns, identify the associated botanical taxa and corresponding phenological stages through palynological analysis, and compare the quantitative yield of three mechanical collector types using linear mixed-effects models.

## 2. Materials and Methods

### 2.1. Study Area and Experimental Apiaries

This study was conducted over two consecutive annual production cycles (2022–2023 and 2023–2024), covering both the dry season (December–May) and the rainy season (June–November) as defined by CONAGUA [10]. The field trial was carried out at two experimental apiaries of the Facultad de Estudios Superiores Cuautitlán (FES Cuautitlán), Universidad Nacional Autónoma de México (UNAM), located at km 2.5 of the Cuautitlán–Teoloyucan highway, San Sebastián Xhala, Cuautitlán Izcalli, State of Mexico: Apiary P-25 (Plot 25; 19°41′50.1″ N, 99°11′23.0″ W) and Apiary CEA (Centro de Enseñanza Agropecuaria; 19°41′24.8″ N, 99°11′52.2″ W) [12,13]. Both apiaries are situated within a disturbed agricultural landscape characterized by mixed herbaceous, ruderal, and cultivated flora, as previously surveyed and detailed by Alcivar-Saldaña et al. [14]. Regional meteorological parameters, including monthly rainfall (mm) and ambient temperature (°C), were compiled from the local meteorological station and validated using historical databases (Weather Spark).

### 2.2. Colony Selection, Standardization, and Experimental Design

The study was conducted across two experimental apiaries (P25 and CEA), each with a total population of 30 *Apis mellifera* colonies in an agricultural, disturbed landscape characterized by mixed herbaceous, ruderal, and cultivated flora, as previously described by Alcivar-Saldaña et al. (2024) [14]. From each apiary, 12 clinically healthy, queen-right colonies compliant with national sanitary regulations, housed in 10-frame Jumbo-type Langstroth hives, were selected (*N* = 24 colonies in total) and monitored longitudinally over 24 consecutive months. At trial initiation, the experimental colonies were equalized and standardized to strong populations consisting of approximately 35,000–40,000 adult worker bees, covering 8 to 9 frames with abundant open and capped brood, as well as balanced honey and pollen reserves. Throughout the two-year experimental period, all colonies received identical routine apicultural management, including the same sanitary inspections (monitoring *Varroa destructor* infestation levels without chemical interventions during propolis collection periods) and equivalent nutritional supplementation during seasonal dearth periods to prevent swarming and minimize colony-level confounding effects. Within each apiary, the 12 selected hives were randomly assigned to one of three propolis harvesting treatments (*n* = 4 colonies per treatment per apiary; *n* = 8 total experimental units per treatment):

- Method 1: Polyurethane collectors [15]
  ▪ Group 1 (G1): Black high-density polyurethane slotted grid collector (slots measuring 2.0 mm × 35.0 mm; 2.0-mm-thick crossbars).
  ▪ Group 2 (G2): Beige high-density polyurethane slotted grid collector (slots measuring 2.0 mm × 35.0 mm; 2.0-mm-thick crossbars).
- Method 2: Flexible mosquito net [15]
  ▪ Group 3 (G3): Flexible thermoformed polyethylene mesh (openings measuring 1.0 mm × 1.0 mm; 0.5-mm-diameter filament).

The individual colony (hive) was considered the experimental unit. We maintained a longitudinal follow-up of the same 24 colonies throughout the two-year study period (2022–2024), with each colony retaining its assigned harvesting method. Collectors were installed directly above the brood chamber at the beginning of each month and retrieved at the end of the month (24 repeated monthly harvests per colony). Propolis was harvested following freezing of the collectors (−18 °C for 24 h) and weighed using a precision balance (Tefal Optiss, Groupe SEB, Écully, France, ±0.01 g precision).

### 2.3. Propolis Harvesting and Palynological Analysis

We maintained a longitudinal follow-up on the same 24 colonies throughout the two-year experimental period. Collectors were installed directly above the brood chamber frames beneath the inner cover at the beginning of each calendar month and retrieved after 30 days (24 consecutive monthly harvests per hive). Upon retrieval, collectors were frozen for 24 h to facilitate clean mechanical removal of the propolis biomass, which was immediately weighed using an electronic precision balance (Tefal^®^ Optiss). Botanical taxa were determined through acetolytic and non-acetolytic palynological extraction of propolis sediment following the method described by Alcivar-Saldaña et al. [14], cross-referencing regional pollen collections and botanical keys [16,17,18,19,20,21,22,23,24,25,26,27,28,29,30,31,32,33,34,35,36,37,38,39,40,41,42,43].

### 2.4. Statistical Analysis

We verified data normality and homoscedasticity using the Shapiro–Wilk and Levene’s tests, respectively. Because monthly observations from the same colonies constitute repeated measures over time and violate the assumption of independence, propolis yield was modeled using a linear mixed-effects model (LMM). The model included Collection Method (G1, G2, G3), Apiary (P25, CEA), Season (Dry, Rainy), and Month as fixed effects, with Hive ID nested within Apiary specified as a random intercept:

$$Y_{ijkl}=\mu +{\mathrm{Method}}_{i}+{\mathrm{Apiary}}_{j}+{\mathrm{Season}}_{k}+{\mathrm{Month}}_{l}+\left({\mathrm{Hive}}_{m\left(j\right)}\right)+\varepsilon_{ijklm}$$

where

- *Y_ijkl_* represents the harvested propolis weight (g);
- *μ* is the overall intercept;
- *M_i_* is the fixed effect of the harvesting method (*i* = G1, G2, G3);
- *A_j_* is the fixed effect of the apiary (*j* = P-25, CEA);
- *S_k_* is the fixed effect of the season (*k* = dry, rainy);
- *T_l_* is the fixed effect of sampling month (*l* = 1, …, 12);
- (*M* × *S*)*_ik_* and (*M* × *A*)*_ij_* are the fixed interaction terms;
- *u_j_*_(*m*)_ is the random intercept of colony *m* nested within apiary *j*;
- *ε_ijkl_* is the residual error.

Degrees of freedom were estimated using the Kenward–Roger approximation, and post hoc multiple comparisons were adjusted using Tukey’s honest significant difference (HSD) test (*α* = 0.05). The bivariate relationship between monthly biomass and cumulative precipitation was assessed using Pearson’s linear correlation (*r*) and simple linear regression (*R*^2^) in R (v4.3.2). Linear mixed-effects models were fitted using the lme4 package; degrees of freedom and *p*-values were estimated using the Kenward–Roger approximation, and post hoc pairwise comparisons were adjusted using Tukey’s honest significant difference (HSD) test via the emmeans package. Bivariate relationships were assessed using Pearson’s linear correlation and simple linear regression.

## 3. Results

### 3.1. Palynological Spectrum and Phenological Calendar

The palynological analysis of the propolis samples identified 23 families, 38 genera, and 39 species, with the herbaceous habit predominating among the identified taxa. *Brassica rapa* was the dominant pollen type, serving as a primary nutritional marker and a complementary balm contributor in this agricultural setting. In contrast, ruderal Asteraceae such as *Bidens* spp. and *Taraxacum campylodes* were the main secondary herbaceous sources. Conversely, resinous and balsamic contributions were primarily linked to dominant arboreal and shrub taxa, including *Eucalyptus globulus*, *Populus* spp., *Pinus* spp., and *Schinus molle*. Identifying these species using specialized botanical literature [14] allowed us to establish their phenological cycles and build a comprehensive propolis-producing vegetation calendar, categorizing taxa by growth habit and function as dominant or secondary pollen and resin sources (Table 1).

### 3.2. Propolis Biomass Yield and Collector Performance

Overall propolis yield averaged 9.02 ± 0.58 g/colony/month (*N* = 576 observations from 24 colonies monitored over 24 consecutive months). Harvested biomass exhibited marked temporal fluctuations governed by seasonal factors rather than collector design.

The linear mixed-effects model (LMM), with colony specified as a random intercept, revealed a highly significant fixed effect for Month (*F*_(11,231)_ = 28.45, *p* < 0.001) and Season (*F*_(1,21)_ = 14.12, *p* = 0.001). Propolis foraging showed a clear bimodal pattern: a primary peak occurred in April across both apiaries (average 20.25 g/colony, with means of 12.25 g in G1, 22.00 g in G2, and 27.00 g in G3), coinciding with vegetative bud burst. Following a sharp summer decline during peak flowering and the zenith of precipitation (6.25 g/colony in July), a secondary increase was recorded in September–October (9.77 g/colony and 9.92 g/colony, respectively), coinciding with the onset of autumnal leaf senescence.

**Figure 1:**
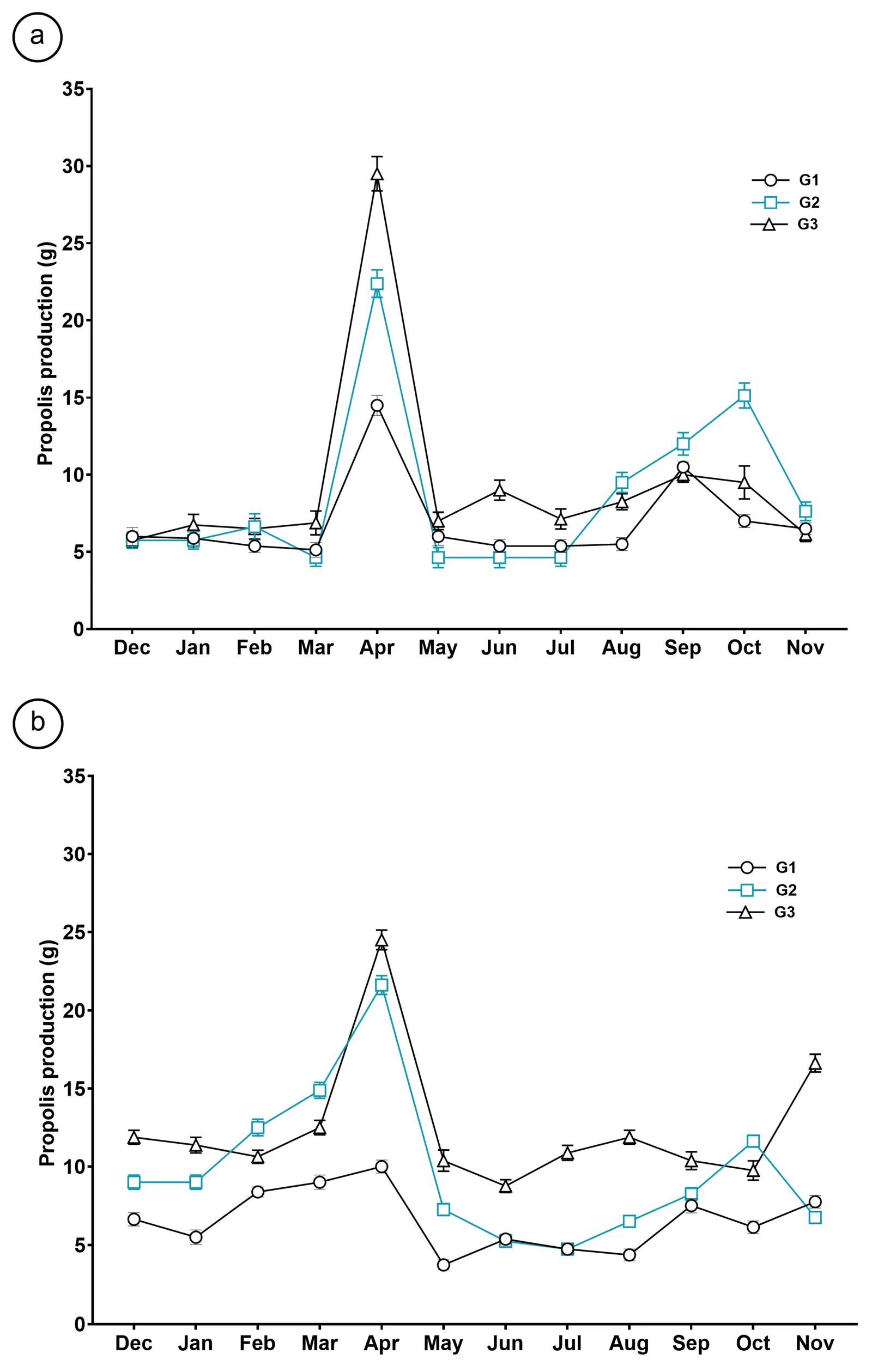
Monthly propolis yield harvested across experimental groups in the Mexican Altiplano over the two-year longitudinal study (2022–2024). (**a**) P25 apiary (overall mean = 8.42 ± 0.85 g/colony/month); (**b**) CEA apiary (overall mean = 9.63 ± 0.96 g/colony/month). Experimental groups: G1 = black polyurethane collector; G2 = beige polyurethane collector; and G3 = flexible polyethylene mosquito mesh (*n* = 4 colonies per treatment per apiary; *n* = 8 total colonies per treatment).

Conversely, Collection Method showed no statistically significant differences (*F*_(2,21)_ = 2.14, *p* = 0.142), with estimated marginal means of 6.88 g for G1, 8.98 g for G2, and 11.21 g for G3. Tukey’s pairwise comparisons confirmed statistical similarity among treatments (G1 vs. G2; G1 vs. G3; G2 vs. G3). The Method × Month interaction was also not significant (*F*_(22,231)_ = 1.08, *p* = 0.368), confirming that the relative performance of the collectors did not depend on the sampling month. Apiary location produced no significant differences (*F*_(1,21)_ = 3.42, *p =* 0.078; P25: 8.42 g/month; CEA: 9.63 g/month) (Table 2, Figure 1).

### 3.3. Phenological Synchronization

**Figure 2:**
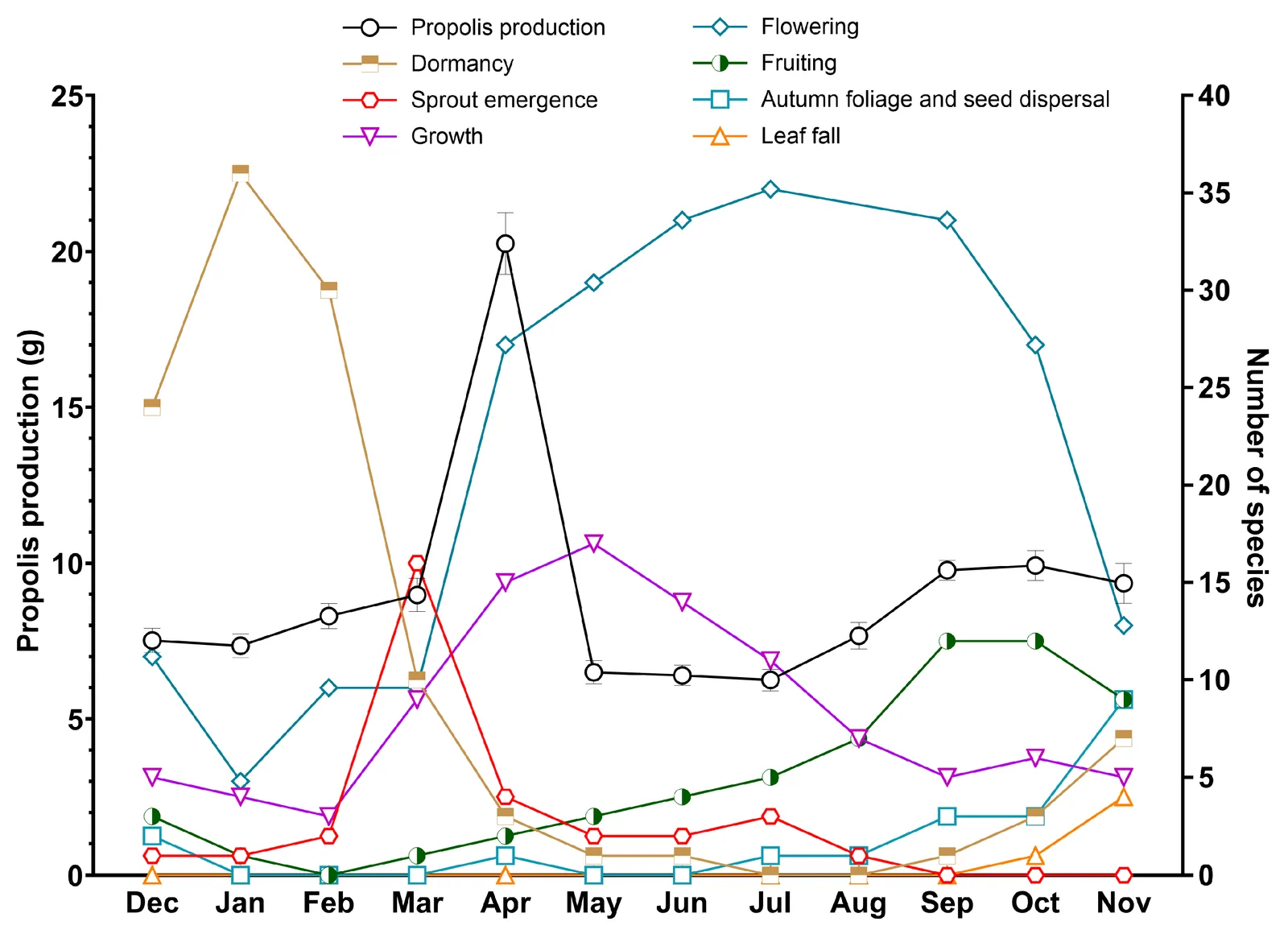
Comparison of phenological cycles of propolis-producing species and propolis production. Values represent monthly means across the two-year monitoring period (2022–2024) and illustrate the bimodal harvesting peaks in April (20.25 ± 1.85 g/colony) and October (9.92 ± 0.73 g/colony).

The analysis of plant phenological cycles in relation to propolis harvesting dynamics revealed that propolis biomass reached a pronounced peak during the massive vegetative bud burst of arboreal species in April (20.25 ± 1.85 g/colony), followed by a marked and statistically significant decline at the onset of mass flowering in May (6.50 ± 0.58 g/colony; Tukey’s HSD test, *p* < 0.001), remaining at minimum levels throughout the period of peak summer vegetative growth and fruiting (July: 6.25 ± 0.54 g/colony). A secondary increase in production was observed during late summer and autumn, coinciding with early defoliation and leaf senescence (September: 9.77 ± 0.61 g/colony; October: 9.92 ± 0.73 g/colony), before decreasing during winter dormancy (Figure 2).

### 3.4. Precipitation Dynamics and Correlation

**Figure 3:**
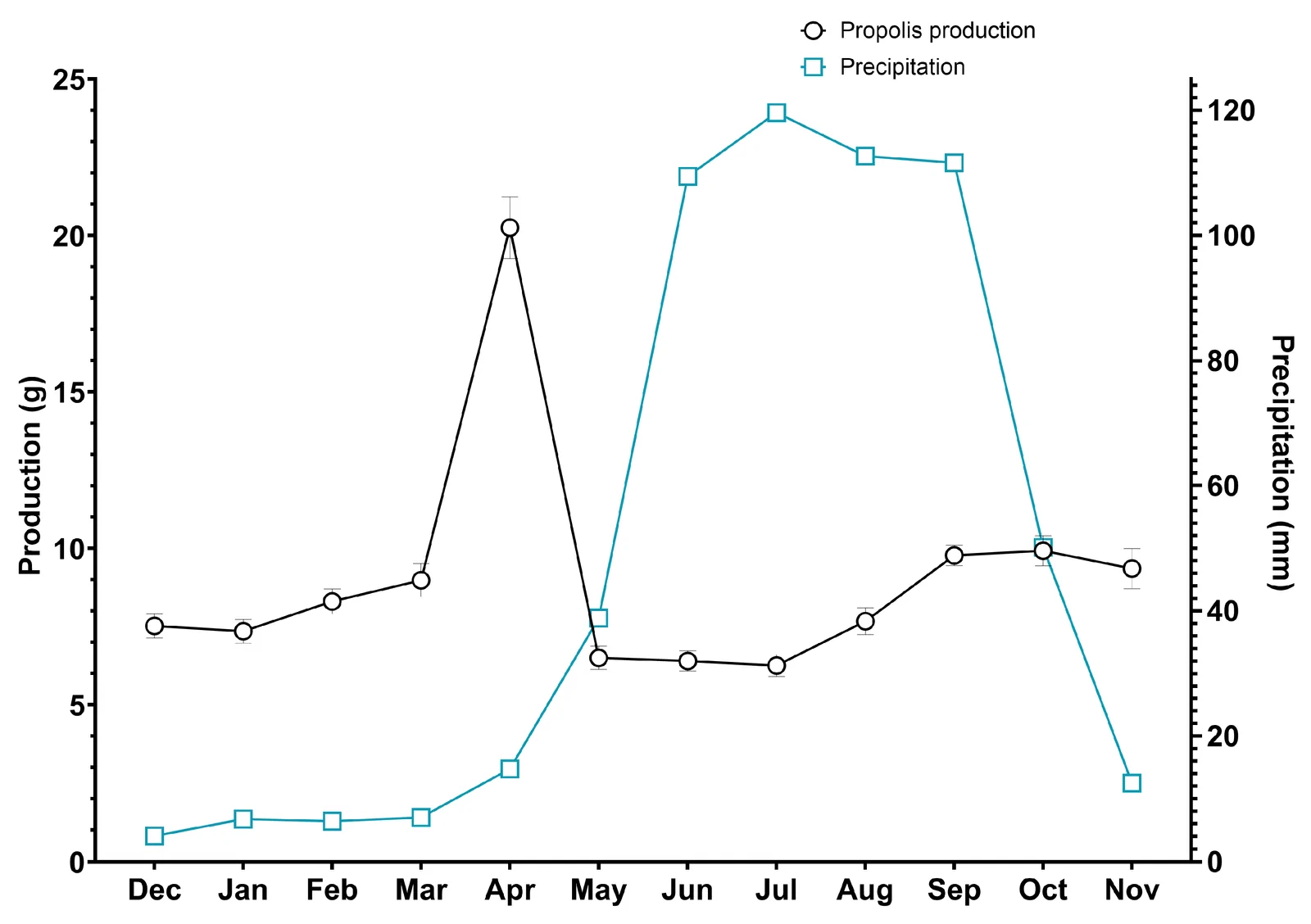
Correlation between propolis biomass production and monthly rainfall in the Mexican Altiplano across the two-year monitoring period (2022–2024). Bimodal peaks are highlighted in April (20.25 ± 1.85 g/colony) and October (9.92 ± 0.73 g/colony).

Cumulative monthly rainfall exhibited a moderate negative linear correlation with propolis biomass, accounting for 17.85% of the linear variance (Figure 3).

## 4. Discussion

This study established a correlation between phenological cycles, precipitation, and propolis production in a beekeeping area of the Altiplano, showing that these variables influence the amount of propolis produced by bees. Furthermore, the identification of species revealed a generalist foraging behavior typical of *A. mellifera* in disturbed neotropical environments or under agricultural conditions [1,6,8,9].

Palynological analysis of the propolis samples identified *Brassica rapa* pollen as the predominant pollen type, a pattern frequently observed in propolis produced in anthropized or agricultural landscapes [44]. However, a critical methodological distinction must be drawn between pollen representing nutritional foraging (nectar and pollen sources) and pollen adhering adventitiously from true resin-secreting plants. Because melissopalynological analysis characterizes airborne or corbicular pollen grains trapped within the hive matrix rather than vegetative resins directly, the predominance of *B. rapa* should not be overinterpreted as evidence of resin production by this herbaceous species. Rather, these and certain ruderal taxa may secrete axillary exudates termed balms that become incorporated into the matrix [8,14,45]. Therefore, in this agroecosystem, *B. rapa* acts primarily as a dominant nutritional marker reflecting surrounding floral availability, whereas co-occurring woody and arboreal taxa such as *Populus*, *Eucalyptus*, *Pinus*, and *Schinus molle overwhelmingly contribute true structural resins*.

Although this study did not directly evaluate the physiological variables or ethological patterns of the colonies, the observed increases in propolis collection and production coincide with key ecological and phenological events previously reported in the literature. Such records document budburst and leaf abscission as physiological processes that enhance the exudation and availability of botanical resins rich in antimicrobial compounds. These resins may be used at the beginning of the season to coat cells internally before queen oviposition. They may also serve as a preventive mechanism linked to hygienic behavior and the thermal and structural conditioning of the nest against winter stress [1,2,3,4]. Consequently, these factors should not be interpreted as mechanisms confirmed by our results, but rather as plausible hypotheses supported by prior evidence that warrant targeted experimental verification in future research.

In turn, the results showed an increase in propolis production during the second half of the dry season, coinciding with the massive sprouting of many plant species [46,47]. This aligns with international studies, particularly those conducted in temperate and subtropical regions, which describe how, during this period, a greater variety of resins with different properties become available; these are easier to collect since they are found in the terminal buds (sprouts) [48,49]. Similarly, the published articles explain that the increase in propolis collection during the rainy season, between September and October, is mainly due to leaf fall. During leaf fall, which is related to the thermal sealing phase, bees repair cracks and gaps in the hive lacking this coating, increasing propolis production to reduce drafts and prepare for winter thermoregulation [50,51].

For the evaluated methods, we found no statistically significant differences in yield under the specific conditions of this study. However, this absence of statistical significance should not be interpreted as evidence that the harvesting method does not influence propolis yields. Rather, our findings suggest that environmental variability—such as resource availability, phenology, and abiotic constraints—exerted a prevailing influence that may have masked potential differences among methods during this trial. As Ribeiro (2009) [52] notes, the amount of propolis harvested is closely linked to colony adaptation to fluctuating botanical sources and ambient conditions. Factors such as precipitation and seasonality directly regulate foraging dynamics and colony homeostatic demands [9,52], indicating that while the mechanical stimulation provided by collection devices remains relevant, its detectable effect can be strongly modulated or overridden by predominant environmental and seasonal pressures.

Meanwhile, the negative correlation observed between propolis production and precipitation (*r* = −0.422, *p* < 0.05) indicates a moderate association, consistent with previous findings on Brazilian propolis where rainfall acts both as a physical constraint on foraging flights and as a factor altering the viscosity and collection of resins and balsams [9]. Although precipitation accounts for approximately of the variance in propolis yield (*R*^2^ = 0.178), the remaining variance (82.2%) reflects the multifactorial nature of harvesting resources essential for this product. While variables such as floral and vegetative phenology, colony strength, and internal demand for resins and balsams may contribute to this variation, these factors were not partitioned in the present analysis and warrant targeted multifactorial modeling in future studies [3,48,49].

In synthesis, the temporal yield patterns observed in the Mexican Altiplano reflect an adaptive resource-allocation trade-off governed by ambient pressures rather than mechanical hive modifications. This opportunity-cost dynamic is empirically demonstrated by our field data when contrasting April and May: propolis collection reached its maximum peak in April (20.25 ± 1.85 g/colony) during vegetative bud burst when few species were in bloom, plummeting significantly in May (6.50 ± 0.58 g/colony; *p* < 0.001) precisely as the landscape transitioned into mass flowering (surpassing 30 blooming species in the phenological spectrum; Figure 2, Table 1). Under optimal foraging theory, this abrupt drop in biomass illustrates an ethological reallocation of worker foragers, who prioritize nectar and pollen over resin collection when abundant floral rewards become available. Conversely, the secondary increase in September–October (9.77 ± 0.61 g and 9.92 ± 0.73 g/colony, respectively) aligns with autumnal defoliation, the cessation of mass flowering, and thermal decline, reflecting a renewed investment in sealing air drafts and social immunity before overwintering. Collectively, these quantitative dynamics confirm that propolis foraging is an environmentally synchronized trade-off driven by floral phenology and colony nutritional priorities, overriding any effect of collector design [49,50,51,52].

## 5. Conclusions

In conclusion, this study shows that the relationship between precipitation and propolis biomass acts as a contributing environmental modulator rather than the primary driver, accounting for about 17.85% of total variance and interacting with broader climatic and botanical phenological factors to shape resource dynamics.

Regarding the harvesting devices, the lack of statistically significant differences in yield suggests that the practical adoption of any method will depend on operational criteria—such as ease of harvesting and handling—rather than productive superiority. Although the commercial collector presented operational advantages during propolis harvesting, specific parameters related to product purity, chemical contamination, or physical residues were not directly quantified in this trial and warrant targeted quality assessments. Furthermore, our palynological findings reflect intense foraging activity on surrounding ruderal and agricultural flora, such as *Brassica rapa*, which serves primarily as a key nutritional and environmental marker in modified agroecosystems rather than a confirmed resin source, leaving true resin contributions to co-occurring woody taxa.

This study has limitations that warrant acknowledgment. First, the two-year observation window may not fully capture long-term interannual climatic variability. Second, melissopalynological analysis served as an indirect proxy for foraging activity and potential botanical origins and does not definitively identify resin exudation sources. Finally, the observational nature of the reported correlations precludes establishing direct causal mechanisms between environmental factors and propolis production. Future research should use controlled multifactorial trials that incorporate comparative chemical profiling, direct foraging observations, and standardized purity evaluations across different collector designs.

## Figures and Tables

**Table 1:** Propolis-producing vegetation identified through palynological analysis, along with their respective phenological cycles.

Calendar of Phenological Cycles of the Propolis-Producing Vegetation																											
Family	Genus	Species	Type	Dry Season												Rainy Season											
				D		J		F		M		A		M		J		J		A		S		O		N	
Altingiaceae	*Liquidambar*	*Liquidambar styraciflua* var. *mexicana* (Oerst.) J.L.Thomas	Deciduous tree																								
Amaranthaceae	*Amaranthus*	*Amaranthus hybridus* L.	Annual herb																								
Anacardiaceae	*Schinus*	*Schinus molle * L.	Evergreen tree																								
Asparagaceae	*Yucca*	*Yucca elephantipes* Regel	Evergreen shrub																								
Asteraceae	*Baccharis*	*Baccharis salicina* Torr. & A. Gray	Evergreen shrub																								
	*Bidens*	*Bidens aurea* (Aiton) Sherff	Rhizomatous perennial herb																								
		*Bidens odorata * Cav.	Annual herb																								
		*Bidens pilosa * L.	Annual herb																								
	*Galinsoga*	*Galinsoga parviflora* Cav.	Annual herb																								
	*Helminthotheca*	*Helminthotheca echioides* (L.) Holub	Annual/Biennial herb																								
	*Simsia*	*Simsia amplexicaulis* (Cav.) Pers.	Perennial herb																								
	*Taraxacum*	*Taraxacum campylodes * G.E.Haglund	Perennial herb																								
	*Tithonia*	*Tithonia tubaeformis* (Jacq.) Cass.	Perennial shrub/Robust perennial herb																								
	*Viguiera*	*Viguiera excelsa* (Willd.) Benth. & Hook.f.	Perennial shrub																								
Brassicaceae	*Brassica*	*Brassica rapa * L.	Annual/Biennial herb																								
	*Raphanus*	*Raphanus raphanistrum* L.	Annual herb																								
	*Sisymbrium*	*Sisymbrium irio* L.	Annual herb																								
Burseraceae	*Bursera*		Deciduous shrub																								
Convolvulaceae	*Ipomoea*	*Ipomoea purpurea* (L.) Roth	Annual climber (vine)																								
Cucurbitaceae	*Sicyos*	*Sicyos deppei* G.Don	Annual climber (vine)																								
Cupressaceae	*Cupressus*	*Cupressus lusitanica * Mill.	Evergreen tree																								
Euphorbiaceae	*Ricinus*	*Ricinus communis* L.	Perennial shrub																								
Fabaceae	*Acacia*	*Acacia retinodes* Schltdl.	Evergreen tree																								
Malvaceae	*Sphaeralcea*	*Sphaeralcea angustifolia* (Cav.) G.Don	Perennial herb/Subshrub																								
Moraceae	*Ficus*	*Ficus retusa* L.	Evergreen tree																								
Myrtaceae	*Eucalyptus*	*Eucalyptus globulus * Labill.	Evergreen tree																								
	*Melaleuca*	*Melaleuca citrina* (Curtis) Dum.Cours.	Evergreen tree/Shrub																								
Oleaceae	*Fraxinus*	*Fraxinus uhdei* (Wenz.) Lingelsh.	Evergreen/Semi-deciduous tree																								
	*Ligustrum*	*Ligustrum japonicum* Thunb.	Evergreen shrub																								
Onagraceae	*Oenothera*	*Oenothera elata* Kunth	Biennial/Perennial herb																								
		*Oenothera rosea* L’Hér. ex Aiton	Perennial herb																								
Pinaceae	*Pinus*		Evergreen tree																								
Poaceae	*Zea*	*Zea mays* L.	Annual herb																								
Polemoniaceae	*Loeselia*	*Loeselia mexicana* (Lam.) Brand	Herb/Subshrub																								
Polygonaceae	*Persicaria*	*Persicaria hydropiperoides* (Michx.) Small	Perennial herb																								
		*Persicaria lapathifolia* (L.) Delarbre	Annual herb																								
Resedaceae	*Reseda*	*Reseda luteola* L.	Biennial herb																								
	*Rosa*	*Rosa canina* L.	Deciduous shrub																								
Salicaceae	*Populus*		Deciduous tree																								
Solanaceae	*Jaltomata*	*Jaltomata procumbens* (Cav.) J.L.Gentry	Perennial herb																								
	*Nicotiana*	*Nicotiana glauca* Graham	Evergreen shrub																								
	*Solanum*	*Solanum elaeagnifolium* Cav.	Rhizomatous perennial herb																								
					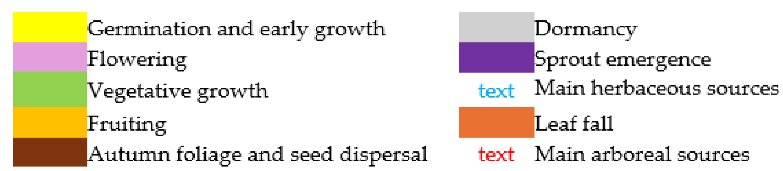																						

**Table 2:** Monthly propolis yield, fixed effects, mean precipitation, and corresponding colony behavioral and phenological phases in the Mexican Altiplano over a two-year monitoring period (2022–2024).

Monthly Propolis Yield						
Month	Monthly Mean (g)	SE (±)	Month Effect	Mean Precipitation (mm)	Dominant Phenological Phase	Biological Behavior of the Colony
**DEC**	7.52	0.78	−1.5	4	Vegetative dormancy	Reduced foraging and winter cluster formation [1,2]
**JAN**	7.35	0.69	−1.67	6.5	Vegetative dormancy	Passive thermal maintenance of the brood nest [1,2]
**FEB**	8.3	0.74	−0.72	6.2	Swollen buds/Transition	Initiation of spring orientation/scouting flights
**MAR**	8.98	0.81	−0.04	7	Shoot emergence	Sanitization and conditioning of the brood area [1,2]
**APR**	20.25	1.85	11.23	14.5	Massive vegetative bud burst	Primary peak: Intensive resin foraging on buds [1,2]
**MAY**	6.5	0.58	−2.52	37	Onset of mass flowering	Foraging shift to nectar/pollen (opportunity cost) [1,2]
**JUN**	6.4	0.52	−2.62	105	Peak flowering/Wet season	Rainfall flight restriction; nutritional prioritization [1,2]
**JUL**	6.25	0.54	−2.77	115	Fruiting/Peak rainfall	Resin dilution and exudate wash-off [1,2]
**AUG**	7.67	0.68	−1.35	108	Late fruiting	Gradual resumption of bark resin collection [1,2]
**SEP**	9.77	0.61	0.75	107	Early defoliation	Foraging on deciduous trees; draft sealing [1,2]
**OCT**	9.92	0.73	0.9	49	Leaf senescence/Seed set	Secondary peak: Social immune envelope expansion [1,2]
**NOV**	9.35	0.95	0.33	12	Dormancy/Flowering cessation	Final hive entrance draft sealing and nest architecture [1,2]

## Data Availability

The original contributions presented in this study are included in the article. Further inquiries can be directed to the corresponding author.
